# Occupational Therapy in Haiti: A Pilot Study to Identify Intervention Methods Used during Short-Term Medical Missions

**DOI:** 10.1155/2020/4198402

**Published:** 2020-08-25

**Authors:** Sheelagh M. Schlegel, Kathleen Mathieson

**Affiliations:** Center of Graduate Health Studies, A.T. Still University, 5850 E Still Cir, Mesa, AZ 85206, USA

## Abstract

Due to the shortage of occupational therapists (OTs) in Haiti and over 800,000 individuals with disabilities, most occupational therapy assessments and interventions are provided by OTs on short-term medical missions (STMMs). Learning which methods OT use to provide assessments and interventions during these STMMs is the first step to understanding how to facilitate follow-up and carry-over for clients and ensure longevity for STMMs in Haiti. This study used a cross-sectional, descriptive design to gather data on methods used by OTs. Thirty-three OTs, who travelled to Haiti on STMMs, completed a 16-question, online survey. The most common method provided by OTs was education to patients, caregivers, and local providers. Training of Haitian rehabilitation technicians was also prevalent. There was an association between the years of the OTs' clinical experience and the effort of OTs to train local providers, but this result was not statistically significant. Further research should be implemented on specific methods that can be used in the absence or shortage of Haitian OTs to ensure follow-up for Haitian clients. The sharing of data regarding OT methods on STMMs will promote evidence-based, client-centered, and cost-effective therapy to enhance effective client outcomes.

## 1. Introduction

Short-term medical missions (STMMs) of one to 30 days duration provide direct medical care to populations in low-income countries (LICs) [1]. Occupational therapy is useful in an area where limited resources are available [2], and occupational therapists (OTs) participate in STMMs to improve clients' independence in their occupations such as work, play, and self-care. Many STMM teams return several times to the same location or liaise with the local providers on a continual basis, but a lack of research into methods that can enhance STMM outcomes exists [3].

Historically, STMMs were faith-based [4], but now many nonprofit organizations or educational institutions sponsor them [5]. STMMs prepare health professionals to be more globally experienced and give them a chance to donate their time and knowledge [1, 6]. Most literature on STMMs, however, is limited to descriptive articles [1, 5, 7]. Only 6% of the articles published on STMMs between 1993 and 2013 involved quantitative or qualitative research [4]. Data collection from most STMMs has focused on quantity, such as the number of patients seen, not health outcomes [3].

STMMs may cause actual harm to communities, exacerbating their existing challenges [3, 5, 7]. Cultural differences may make communication difficult, and providers may be unfamiliar with the local culture and health care systems, rendering them unable to provide quality and effective care [1, 8]. Donated supplies and equipment may fulfill short-term needs for a community but may cause long-term supply issues [3]. Donations can also cause harm if not supplied correctly, may threaten the livelihood of local providers, and may increase the financial demand for parts and maintenance on communities who are already struggling [9]. As stated by Decamp et al. [10], STMMs must focus on prevention, educating local providers, and building health care infrastructure to facilitate long-term change. STMMs that provide short-term benefits, such as care after humanitarian crises, can evolve into long-term partnerships [10]. This is certainly the case in Haiti, where many occupational therapy teams return annually or semiannually to follow-up with clients and engage in long-term planning.

Haiti, the poorest country in the Western hemisphere, has a population of 9 million people. An estimated 800,000 people are living with disabilities in Haiti, and between 194,000 and 250,000, people were injured in the 2010 earthquake [11]. Haitian individuals with disabilities experience vulnerabilities due to difficulty meeting basic needs, safety concerns, and lack of access to health care and other services [12]. A severe shortage of rehabilitation professionals exists in Haiti with only 30 physical therapists and six OTs working in the country [13, 14]. Haiti Rehabilitation Foundation, a nonprofit organization, established an OT baccalaureate training program at Faculté des Sciences de Réhabilitation de Léogâne (FSRL) in Haiti in 2015 and the first class of three OTs graduated in early 2020 [15]. Although rehabilitation services have increased in Haiti since the 2010 earthquake and there has been the institution of the new occupational therapy program, the shortage of rehabilitation professionals is severe.

Due to the lack of of OT practitioners in Haiti, several organizations send therapists on STMMs to Haiti to provide direct care, consultation, and training for local providers. Several organizations have become adept at long-term planning and ensuring follow-through for OT clients during and in between STMMs [16, 17]. To ensure that occupational therapy programs are sustained, STMM teams have implemented methods such as training community health workers or rehabilitation technicians, educating clients and their families, and using telerehabilitation.

Community health workers (CHWs) have been used for many years in Haiti and can be an effective means of providing health education in a culturally-appropriate and meaningful way where there are no health professionals [18]. Rehabilitation technicians are also used widely in Haiti [14]. They can work with clients on a range of motion exercises, adaptive equipment, feeding and swallowing techniques, and mobility programs under the intermittent supervision of a therapist [17].

Although there is no literature linking it to Haiti, educating a client or a caregiver is an essential method used by OTs and tailored to an individual's needs [19]. Coaching is a strength-based method, aligned with family-centered care [20] and when coaching, OTs guide clients with deficits in functional performance to reach their goals [21]. The teach-back method is widely used by providers to check if clients or caregivers can understand and describe in their own words about their health or demonstrate what they need to know [22]. Caplin and Saunders [23] reported that the teach-back method is effective for OTs to use to educate clients because it ensures client understanding in between OT visits and reduces the risk that clients with low literacy do not understand what is being taught.

One method that is often used in a global setting to provide services where there are no providers is telehealth. Telehealth provides consultative services to remote and rural areas using videoconferencing, and cell phones with systems designed to work with limited broadband and hardware that are available in a LIC [24, 25]. Telehealth is often associated with improved clinical decision-making and increased efficiency for health care providers and is a method that could be used in a global setting, like Haiti where there is a shortage of providers. The use of remotely-supervised telerehabilitation by OTs can enhance client experiences, empower clients, and decrease cost [26].

Part of the vision of OT is to provide culturally-responsive and customized services and to provide evidence-based, client-centered, and cost-effective therapy to enhance effective outcomes for clients [27]. Continuity and training methods are necessary to ensure that OT programs are sustained. With the shortage of qualified OTs in Haiti, STMMs are the only way to augment the limited number of therapists. However, a gap in the literature exists regarding methods that can help improve the carry-over of OT interventions. The purpose of this cross-sectional, descriptive study was to determine what methods OT teams use to provide interventions during and after STMMs to Haiti as a first step in understanding how to ensure follow-up and carry-over for clients and facilitate longevity for STMMs in Haiti.

## 2. Materials and Methods

This study was a cross-sectional descriptive study, and the variables of interest were demographics of participants and the type of methods used to provide follow-up and carry-over for clients who received OT. Study participants completed an online, anonymous survey. To be included, participants had to be OTs with a bachelor's degree or higher who participated in at least one STMM to Haiti. OT students who participated in service learning trips to Haiti and OTs who volunteered in Haiti for purposes other than providing direct OT services were excluded. The A. T. Still University Institutional Review Board approved the study.

The first author developed a survey that included items to gather demographic information and items to gather data on the methods OTs use on STMMs to ensure follow-up and carry-over for the clients between trips Table 1. The demographic data collected on the OTs included their age, country of origin, educational level, years of experience, and the number of STMMs the OTs had participated in. These methods included those found in the literature, such as telerehabilitation, education methods, and training of rehabilitation technicians and community health workers.

At the end of the survey, two open-ended questions asked participants to describe their challenges to providing follow-up for clients between STMMs and asked them to outline other methods they used to ensure long-term benefits. The investigator designed these open-ended questions to provide some information to highlight or provide examples of the main quantitative findings from the closed-ended questions. The survey was beta-tested by three content experts, an outside reviewer, and the subinvestigator. Revisions were made, and the survey was administered using the Survey Monkey platform.

The first author contacted the Haitian Association of Occupational Therapists, nonprofit organizations, and the Dean of the FSRL occupational therapy program and asked each to forward an email to OTs who participated in STMMs to Haiti. This email had an embedded link that connected participants to the electronic survey. Other participants were referred by OTs using the snowball sampling method. The use of convenience sampling may have limited the ability to generalize the results to the target population of OTs who participate in STMMs in Haiti but as there are no official records of STMMs, this was the only method that was feasible.

The survey was 16 questions and took an average of seven minutes to complete. The investigator collected data over six weeks and sent reminder emails every seven days during the collection period informing potential participants of the closing date of the survey. All survey data collected were anonymous and answers were confidential.

Once the survey ended, the investigator downloaded the data from Survey Monkey into Microsoft Excel and imported it into IBM SPSS Statistics version 25 for analysis. The data collected were at the nominal and ordinal levels. Calculations of frequencies and percentages were conducted, and figures and tables were used to compare types of clients seen and methods used by OTs. A chi-square analysis was conducted to see if there was an association between the number of STMMs that OTs had participated in and their training of local health providers as experts recommend that STMMs must focus not just on providing direct care but also on sustainable practices like education of community stakeholders [10].

## 3. Results and Discussion

### 3.1. Results

A total of 34 respondents completed the anonymous, online survey. The response rate for the survey was 71%. One respondent did not meet the inclusion criteria for the study as they were not an occupational therapist, so their data were excluded from the results. Therefore, there were 33 surveys available for analysis.

The respondents' demographic data and characteristics are summarized in Table 2. The majority of respondents were female (32) with only one male respondent. Most of the respondents were White/Caucasian (94%), followed by Black (3%), and American Indian/Alaskan Native (3%). The majority resided in the United States (63.5%) followed by Canada (27.3%), South Africa (3.0%), and Haiti (3.0%).

The respondents' professional characteristics are also summarized in Table 2. The percentage of the OTs who had a master's degree was 45.5%. Twelve percent had bachelor's degrees, and 15.2% had clinical doctorates in OT (Table 2). One respondent had a PhD in Rehabilitation Science. The majority of the respondents (56.3%) had more than 20 years of experience working as OTs, 6.1% had 16 to 20 years of experience, 6.1% had 11 to 15 years, 9.1% had 6 to 10 years, and 18.2% had 1 to 5 years of experience. Three percent of respondents had less than one year of experience as an OT (Table 2).

The majority of respondents had participated in 2 to 4 STMMs (57.6%), followed by 1 STMM (18.2%) and 5 to 10 STMMs (15.2%), while 9.1% of respondents had participated in more than 10 STMMs (Table 1). An equal number of OTs (39.4%) participated in STMMs sponsored by nongovernmental organizations and by faith-based organizations, 21.2% participated in STMMs sponsored by universities, 15.2% by private organizations, and 18.2% organized their own STMMs (Table 2).

OTs provided interventions to various client populations in Haiti including outpatient pediatric clients (66.7%), outpatient adult clients (54.5%), inpatient pediatric clients (33.3%), home health clients (33.3%), inpatient adult clients (21.2%), and outpatient mental health clients (3%) (Table 3). Ten OTs (30%), who checked the other category for client populations, identified working with children in residential settings (Table 3).

As displayed in Table 4, 28 OTs (84.8%) used patient education as a method, 27 (81.8%) used the caregiver education method, and two OTs (6.1%) used telehealth methods. When it came to the training of others as a method to ensure carry-over, 21 OTs (63.6%) trained local providers, 19 (57.6%) trained rehabilitation technicians, nine (27.3%) trained community health workers, and eight OTs (24.2%) trained other STMM teams.

Within the patient and caregiver education method, 20 OTs (60.6%) used coaching, 19 (57.6%) used home exercise programs, 17 (51.2%) used education materials in Kreyol, and 13 (39.4%) used the teach-back method to educate clients and their caregivers (Table 4). Within the telehealth method, three OTs (9.1%) used phone or video calls with providers, two (6.1%) used mobile health, one used video conferencing, and one used video store-and-forward to ensure carry-over for clients (Table 4).

The training methods that were used by respondents who trained rehabilitation technicians and community health workers are summarized in Table 5. The most common training methods for rehabilitation technicians, used by 26 respondents (78.8%), were training positioning of clients and adaptive equipment, the common training activities. For those respondents who trained community health workers, respondents were also more likely to provide training on positioning (45.5%) and adaptive equipment (39.4%).

A subanalysis was conducted to explore whether OTs, who had increased years of experience, were more likely to use a method like education of local providers to ensure sustainability. Although 75% of OTs with six or more years' experience provided education to local providers versus only 50% of OTs with 1 to 5 years' experience, according to a chi-square analysis, the results were not statistically significant, *χ*^2^ (1, *N* = 32) = 1.57, *p* = 0.21 (Table 6).

For the two open-ended questions at the end of the survey, the most common theme for the question regarding barriers during STMMs was the language barrier (45.5% of respondents). One OT commented that “language barriers can result in difficulty with relaying proper education.” Another theme identified by eight OTs (24.2% of the respondents) was that length of their volunteer experiences in Haiti were too short. For example, one OT commented there was “limited amount of time available with each patient secondary to the number of patients in need of services and short duration of trip,” and another commented that “short duration of the therapist's stay contributed to interrupted rehab services.” Two other themes identified as barriers were lack of local staff in Haiti and lack of resources, both of which were noted by 21.2% of respondents.

On the open-ended question about other methods OTs used, the most common method used was documentation, both to other STMM teams and local providers, to ensure follow-up. Five OTs (15.2%) mentioned using documentation as another method to ensure carry-over and follow-through for clients, and comments included, “I left simple report notes for the center staff and for therapists that followed me” and “I left written communication through an OT note on the treatment I provided and recommendations for follow-up interventions”.

### 3.2. Discussion

The primary purpose of this study was to identify the methods that occupational therapists (OTs), who travel to Haiti on short-term medical missions (STMMs), use to ensure follow-up and carryover for their patients. Most of the study participants were from the United States, followed by Canada. This correlates with a systematic review by Martiniuk et al. [1], who found that most STMM participants were citizens of high-income countries, usually the United States and Canada. The participants in this study were mostly female, which correlates with data from American and Canadian studies where females were represented in the OT workforce as 98% and 90.4%, respectively [28, 29]. The majority of the OTs in the study worked at outpatient clinics in Haiti. This aligns with a study by Parker et al. [30], who found that much of the rehabilitation services in Haiti are provided in clinics set up by nongovernmental organizations due to the Haitian government's lack of ability to provide rehabilitation services.

The most common method used by OTs was patient education, followed by caregiver education, local provider training, and rehabilitation technician training. When examining the survey results for education, the most common method used was coaching, followed by home programs in Kreyol, and use of the teach-back method to educate clients and their caregivers.

Another method used by OTs in this study was the training of rehabilitation technicians. This finding supported studies that showed the importance and availability of rehabilitation technicians or aides and the presence of several training programs in Haiti to provide services in the absence of Haitian therapists [14, 31]. The low utilization of telehealth methods by the OTs in the study may indicate insecurity with using this newer method, or it may be due to a lack of resources in Haiti, which was identified as a barrier by the study participants. Lee et al. [25] found that a good Internet connection with a low but stable bandwidth is necessary for telehealth, but Laguerre [32] noted that Haiti often lacks stable Internet. In addition, not enough technicians are available to assist with hardware or software issues in Haiti and often the electricity supply is unstable [32].

OTs identified the language barrier as the most noted challenge to providing methods for carry-over in Haiti. Garbutt et al. [33] found that a significant barrier between clients and volunteer OTs in low-income countries is a lack of shared language, which is needed for communicating about the services provided. In the current study, another barrier identified by the OTs was the lack of resources in Haiti. Another study by Duggan et al. [17] also found that the lack of material, human resources, and infrastructure in Haiti challenged OTs' flexibility, creativity, and adaptability on STMMs.

## 4. Conclusions

This study had a small sample size (*n* = 33) that limited the generalizability of the results. This small sample size may limit the generalizability of the results, but as the number of OTs who volunteer in Haiti is probably quite small due to the lack of infrastructure and rehabilitation facilities, the sample size may represent the target population.

Study participants were recruited using convenience sampling by contacting OTs from different organizations, as was the snowballing method when it was requested that OTs send the survey to others. This use of nonprobability sampling means that the findings cannot be generalized to the larger population of OTs. In addition, OTs were asked to forward the survey to other OTs, and this may have caused a discrepancy in the known total of questionnaire recipients. The sample represented more females than males (32 : 1) as is usually the case in the profession of OT and a majority of Caucasian study participants. The lack of gender and ethnic diversity is similar to another study of OTs involved in STMMs [33].

Another limitation was that OTs could check more than one category on questions about which methods they used. This may have affected the study results, as respondents may have selected options at the beginning of the list of methods without carefully reading the remaining items and also may have selected other methods than the ones that actually applied to them. According to Mersdorf [34] with check-all-that-apply questions, alternatives may be selected that do not apply or apply only minimally, whereas more important alternatives are not chosen. To address this, the number of responses can be restricted so that respondents prioritize the most important methods [34].

Some survey questions seemed to have caused confusion and may have required further clarification. For example, for the first question regarding an overview of methods used, only 19 OTs checked that they participated in the training of rehabilitation technicians, but on the question pertaining to specific training methods, 26 OTs responded that they had trained rehabilitation technicians in certain methods. There were similar results on the CHW training and telehealth methods, where there were discrepancies between questions about the use of methods.

With the changes in the rehabilitation sector in Haiti due to the new training program for Haitian OTs, conducting further investigation to research how services for clients and the opportunities for OTs to participate in STMMs change over time would be prudent. Future studies could compare different regions of Haiti, such as urban and rural, to evaluate the different types of challenges that arise for OTs providing interventions and seeking follow-up for clients during and in-between STMMs.

Further research should be implemented on specific methods that can be used in the absence or shortage of Haitian OTs to ensure follow-up. Future studies using experimental designs could compare methods such as patient education and educating local providers or to a control group. Other possibilities could include qualitative research studies to interview OTs and their clients regarding methods used to improve sustainability and outcomes for future Haitian clients, their caregivers, and their communities.

It has been estimated that there are over 6,000 short-term medical missions (STMMs) annually around the world, with thousands of volunteers and costs in excess of $250 million [35]. Historically, STMMs have focused mainly on the delivery of services, like surgeries, and this is reflected in the literature. In the past 15 years, the focus has shifted from delivery of care to ethical considerations [36] but there is a lack of research on outcomes from STMMs. Sykes [4] found that the focus of most literature on STMMS has been on quantity, like numbers of patients seen, versus quality data like patient outcomes. Any articles on OT STMMs to Haiti have been descriptive or anecdotal in nature and lacked data collection or theoretical analysis of the care provided.

By simply providing direct care and not sharing information about the methods they used to ensure carry-over for the clients, occupational therapists who have volunteered in Haiti may not be following the current guidelines for STMMs, which are to develop long-term relationships, train local providers, and improve local infrastructure to ensure sustainability, and not to merely provide direct care or donated supplies [3]. This study was the first step in discovering what methods OTs are actually using to improve sustainability and to share this information with OTs who have participated in STMMs to Haiti. This sharing of ideas will promote evidence-based, client-centered, and cost-effective therapy to enhance effective outcomes for Haitian OT clients.

## Figures and Tables

**Table 1:** Survey Questions.

Question
What is your gender?
Which race/ethnicity best describes you?
In which country do you currently reside?
What is your highest degree attained in OT?
How many years of experience do you have as an OT?
Which of the following categories best describes your employment status?
How many short-term medical missions to Haiti have you participated in?
What type of organization(s) coordinated your STMM(s) to Haiti?
What populations did you provide interventions and assessments for during STMMs to Haiti?
During or after your STMM(s) to Haiti did you engage in any of the following activities to ensure follow-up and carry-over for the clients?
If you engaged in patient education or caregiver education to ensure follow-up and carry-over for your clients during or after your STMM, please check which specific education methods you used
If you engaged in telehealth methods during or after STMMS to ensure follow-up and carry-over for your clients, please check which specific methods you used
If you engaged in training of rehabilitation technicians during or after your STMMs to Haiti to ensure follow-up and carry-over for patients please check the specific training activities that you used
If you engaged in the training of community health workers during or after your STMMs to Haiti to ensure follow-up and carry-over for patients, please check the specific training methods that you used
In your experience on a short-term medical mission to Haiti, what are the challenges to providing follow-up and carry-over for patients during or between trips?
Did you provide any other methods, besides those previously mentioned to ensure follow-up and carry-over for clients in between STMMs to Haiti? If yes, please outline the methods below.

*Note:* STMM = short-term medical mission.

**Table 2:** Demographic and professional characteristics of study participants (*N* = 33).

Characteristic	Number	Percentage
Gender		
Female	32	97.0
Male	1	3.0
Residence		
United States	21	63.5
Canada	9	27.3
Haiti	2	6.1
South Africa	1	3.0
Highest degree		
Master's	15	45.5
Bachelor's	12	36.4
Clinical doc	5	15.2
PhD	1	3.0
Years of OT experience		
<1 year	1	3.0
1-5 years	6	18.2
6-10 years	3	9.1
11-15 years	2	6.1
16-20 years	2	6.1
>20 years	18	54.5
No response	1	3.0
No. of STMMs		
1 STMM	6	18.2
2-4 STMMs	19	57.6
5-10 STMMs	5	15.2
>10 STMMs	3	9.1
STMM Organization^a^		
NGO	13	39.4
Faith-based	13	39.4
University	7	21.2
Private	5	15.2
Self-organized	6	18.2

Note: AI/AN: American Indian/Alaskan Native, AA: African American, STMM: short-term medical mission, NGO: nongovernmental organization. ^a^Types of STMM organization do not equal *N* = 33 because respondents were able to choose multiple categories.

**Table 3:** Client populations seen by OTs on STMMs (*N* = 33).

Type of Client^a^	Number	Percentage
Outpatient adult	18	54.5
Outpatient pediatric	22	66.7
Inpatient pediatric	11	33.3
Inpatient adult	7	21.2
Home health	11	33.3
Outpatient mental health	1	3.0

Note: ^a^type of client does not equal *N* = 33 because respondents were able to choose multiple categories.

**Table 4:** Methods used on STMMs (*N* = 33).

Method^a^	Number	Percentage
Education		
Patient education	28	84.8
Caregiver education	27	81.8
Local provider education	21	63.6
Coaching	20	60.6
Teach back	13	39.4
Home exercise programs	19	57.6
Kreyol materials	17	51.5
Telehealth		
Video conferencing	1	3.0
Store and forward	1	3.0
Mobile health	3	9.1
Phone/video calls	3	9.1
Training		
Training of rehab techs	19	57.6
Training of CHWs	9	27.3
Training of other STMMs	8	24.2

Note: ^a^method does not equal *N* = 33 because respondents were able to choose multiple categories. CHWs: community health workers.

**Table 5:** Training for rehabilitation technicians and CHWs (*N* = 33).

Training	Trained RTs	Percentage	Trained CHWs	Percentages
Positioning	26	78.8	15	45.5
Adaptive equipment	26	78.8	13	39.4
Range of motion	23	69.7	12	36.4
Home ex. programs	18	54.5	7	21.2
Mobilization	17	51.5	10	30.3
Making A.E.	16	48.5	5	15.2
Swallowing	12	36.4	7	21.2
Relaxation	3	9.1	2	6.1

Note: ^a^training does not equal *N* = 33 because respondents were able to choose multiple categories. Home ex.: home exercise. A.E: adaptive equipment.

**Table 6:** Years of OT experience vs. education of local providers (*n* = 32).

	Did not educate local providers	Educated local providers
1-5 years OT experience	5 (50%)	5 (50%)
6+ years OT experience	6 (27.3%)	16 (72%)

Note: results reported as row n (%). *χ*^2^(1, *N* = 32) = 1.57, *p* = 0.21.

## Data Availability

The availability of the data is restricted, because, due to the small sample size, there are ethical and confidentiality concerns with providing access to the full, non-aggregated data.
